# Os desafios da continuidade da vacinação contra a COVID-19 em um cenário de hesitação vacinal

**DOI:** 10.1590/0102-311XPT210924

**Published:** 2026-02-13

**Authors:** Vick Brito Oliveira, Adriana Falangola Benjamin Bezerra, Sydia Rosana de Araujo Oliveira

**Affiliations:** 1 Instituto Aggeu Magalhães, Fundação Oswaldo Cruz, Recife, Brasil.; 2 Universidade Federal de Pernambuco, Recife, Brasil.

**Keywords:** Hesitação Vacinal, Vacinas contra COVID-19, Vacinação, Vaccination Hesitancy, COVID-19 Vaccines, Vaccination, Vacilación a la Vacunación, Vacunas contra la COVID-19, Vacunación

## Abstract

Este trabalho objetivou mapear controvérsias relacionadas à interação da descontinuidade do esquema vacinal contra a COVID-19 e a hesitação vacinal, em um município de Pernambuco, Brasil. A pesquisa foi orientada pela cartografia das controvérsias, perspectiva metodológica ancorada na Teoria Ator-Rede. Realizou-se entrevistas com 36 adultos e idosos, com esquema vacinal incompleto, oito profissionais e cinco gestores de saúde, além da confecção do diário de campo para a produção dos dados. Foram identificados três perfis de usuários: confiantes, hesitantes e vacinados por exigência. Vinte e um usuários relataram a intenção em não prosseguir com a vacinação, caso fosse recomendado. Agrupamentos temáticos subsidiaram mapear três controvérsias: a disputa acerca da confiança no cenário da vacinação contra a COVID-19, a disputa sobre a informação, e as disputas na ampliação da cobertura vacinal diante das barreiras de acesso à vacinação. Entre os resultados, destacaram-se que a adesão vacinal não se configurou sinônimo de confiança na vacinação, crenças e sentimentos como confiança e medo estiveram atrelados às experiências pessoais, notícias falsas e debates políticos e ideológicos, com características distintas entre os perfis. A responsabilidade coletiva apresentou-se como importante elemento para o fortalecimento da vacinação, sendo compreendida em articulação com a superação das barreiras vacinais. O estudo apontou a diversidade de atores que atravessaram a descontinuidade do esquema vacinal contra a COVID-19, indicando a necessidade de políticas de aceitação da vacinação, com o fortalecimento da confiança vacinal e, consequentemente, ampliação da adesão e cobertura vacinal.

## Abstract (en)

This study aimed to map controversies related to the interaction of the discontinuity of the vaccination schedule against COVID-19 and vaccine hesitancy in a municipality in the state of Pernambuco, Brazil. This research was guided by the cartography of controversies, a methodological perspective anchored in Actor-Network Theory. Interviews were conducted with 36 adults and older adults with an incomplete vaccination schedule, eight healthcare providers, and five health managers. A field diary was used to produce data. This study found three user profiles: confident, hesitant, and vaccinated by demand. In total, 21 users reported refusing to proceed with vaccination if recommended. Thematic groupings mapped three controversies: disputes over confidence in the COVID-19 vaccination scenario, information, and the expansion of vaccination coverage in the face of barriers to its access. The results highlighted that vaccine adherence was not synonymous with confidence in vaccination and that beliefs and feelings such as trust and fear were linked to personal experiences, fake news, and political and ideological debates, with distinct characteristics between the profiles. Collective responsibility would constitute an important element to strengthen vaccination and overcome vaccine barriers. This study showed the diversity of actors who experienced the discontinuity of the vaccination schedule against COVID-19, indicating the need for policies toward vaccination acceptance, the strengthening of vaccine confidence, and the expansion of adherence and vaccination coverage.

## Abstract (es)

Este trabajo tuvo como objetivo mapear las controversias relacionadas con la interacción entre la discontinuidad del esquema de vacunación contra la COVID-19 y la vacilación vacunal en un municipio de Pernambuco, Brasil. La investigación se basó en la cartografía de las controversias, una perspectiva metodológica basada en la Teoría Actor-Red. Se realizaron entrevistas a 36 adultos y ancianos con vacunación incompleta, ocho profesionales y cinco gestores sanitarios, y se elaboró un diario de campo para la recopilación de datos. Se identificaron tres perfiles de usuarios: confiados, indecisos y vacunados por obligación. Veintiún usuarios manifestaron su intención de no vacunarse, en caso de que se les recomendara. Las agrupaciones temáticas permitieron identificar tres controversias: la disputa sobre la confianza en el escenario de la vacunación contra la COVID-19, la disputa sobre la información y las disputas sobre la ampliación de la cobertura vacunal ante las barreras de acceso a la vacunación. Entre los resultados, se destacó que la adhesión a la vacunación no era sinónimo de confianza en la vacunación, sino que creencias y sentimientos como la confianza y el miedo estaban vinculados a experiencias personales, noticias falsas y debates políticos e ideológicos, con características distintas entre los distintos perfiles. La responsabilidad colectiva se presentó como un elemento importante para el fortalecimiento de la vacunación, entendiéndose en relación con la superación de las barreras a la vacunación. El estudio señaló la diversidad de actores que atravesaron la discontinuidad del esquema de vacunación contra la COVID-19, indicando la necesidad de políticas de aceptación de la vacunación, con el fortalecimiento de la confianza en las vacunas y, en consecuencia, la ampliación de la adhesión y la cobertura vacunal.

## Introdução

O movimento fluido entre o aceite e a recusa vacinal pode apresentar diversas configurações com elementos individuais, grupais, comportamentais, contextuais, quais a literatura vem se debruçando e apresentando possíveis modelos explicativos, dimensões, determinantes e/ou motivadores relacionadas à aceitação, hesitação e recusa à vacina ^1^ ^,^ ^2^ ^,^ ^3^ ^,^ ^4^ ^,^ ^5^ ^,^ ^6^.

Na pandemia da COVID-19, o fenômeno da hesitação vacinal tensionou, desestabilizou e fomentou novas redes sociotécnicas no campo da vacinação, com interações entre atores humanos e não humanos ^7^, cenário este de interesse para a saúde pública. Estudos destacaram a dificuldade de mensurar a hesitação diante das barreiras de acesso, do desafio de lidar com a desinformação e com as consequências de um sentimento de fadiga pandêmica e vacinal ^2^ ^,^ ^8^ ^,^ ^9^ ^,^ ^10^.

No Brasil, a campanha da imunização da COVID-19 transitou no território acompanhado das dificuldades técnicas, alastramento das notícias falsas, disputas político-ideológicas, iniquidades territoriais engendradas por marcadores sociais de diferença e desigualdade ^11^ ^,^ ^12^ ^,^ ^13^ ^,^ ^14^. O cenário esteve entrelaçado à expansão da hesitação vacinal, desenhando obstáculos ao Programa Nacional de Imunizações (PNI).

A partir dessas considerações, este trabalho interessou-se pelo debate desse fenômeno, especificamente em pessoas que iniciaram a vacinação contra a COVID-19 e não completaram o esquema vacinal disponível. Para isso, assumiu-se a premissa que a hesitação se conformou em um cenário marcado por conflitos e disputas, compreendido como um campo de controvérsias, com atores que interagiram em rede ^15^.

Neste sentido, este artigo apresenta os resultados da pesquisa sobre as relações entre a hesitação vacinal e a descontinuidade do esquema COVID-19, com foco na população maior de 18 anos. O debate proposto apresenta controvérsias mapeadas a partir da inserção em unidades de saúde de um município pernambucano.

## Método

A pesquisa caracterizou-se como estudo de caso de caráter exploratório ^16^ realizado em um município da Região Metropolitana do Recife, Pernambuco, utilizando a ferramenta da cartografia das controvérsias ^17^ ^,^ ^18^, ancorada na Teoria Ator-Rede (TAR) ^7^. A TAR se interessa pela descrição dos agenciamentos que constituem a dinâmica social, desestabilizam saberes consolidados e geram conflitos, disputas ^19^. Assim, as alianças, conexões entre os actantes (agenciamentos entre atores humanos e não-humanos) deixam rastros, performam redes sociotécnicas e as controvérsias se mostram ^7^.

Neste estudo, as controvérsias foram mapeadas a partir da ida à quatro unidades de saúde da família (USF) e três policlínicas, sendo uma unidade em cada região sanitária do município, além da visita à Secretaria Municipal de Saúde (SMS). As unidades foram indicadas pela gestão municipal com base no maior fluxo de pessoas vacinadas em serviços com oferta do imunizante e na diversidade do perfil sociodemográfico dos territórios municipais.

Duas USF e uma policlínica estavam localizadas em bairros com acesso facilitado, ao considerar à SMS. As USF apresentaram menor fluxo de pessoas comparada às policlínicas, aspecto esperado, visto que as policlínicas reúnem uma maior oferta de especialidade de serviços médicos, funcionando como centros de saúde para as regiões.

Entrevistas não-estruturadas foram realizadas entre janeiro e junho de 2024 com oito profissionais (P) de saúde e cinco gestoras (G) vinculadas ao Programa Municipal de Imunização (PMI) (Quadro 1). Trinta e seis usuários foram entrevistados, no mesmo período, com o esquema vacinal incompleto (doses disponibilizadas até dezembro de 2023 - mínimo de D1 e D2, dose de reforço e reforço bivalente). Na abordagem para a realização da entrevista, solicitou-se a informação sobre a quantidade de doses recebidas para a COVID-19.

**Quadro 1:** Quadro síntese das características das profissionais e gestoras de saúde entrevistadas. Pernambuco, Brasil, 2024.

ID.	RAÇA/COR	PROFISSÃO/FORMAÇÃO	ESCOLARIDADE	TIPO DE VÍNCULO	TEMPO DE ATUAÇÃO NO SUS	ESQUEMA VACINAL
1	Parda	ACS	Médio completo	Estatutário	27 anos	Atualizado
2	Branca	Enfermeira em USF	Superior completo	Estatutário	7 anos	Desatualizado
3	Branca	Enfermeira em policlínica	Superior completo	CLT	9 meses	Desatualizado
4	Parda	Técnico de Enfermagem em USF	Superior completo	Estatutário	2,5 anos	Atualizado
5	Branca	ACS	Superior completo	Estatutário	7 anos	Atualizado
6	Parda	Técnico de Enfermagem em USF	Médio completo	CLT	10 anos	Atualizado
7	Preta	Auxiliar de Enfermagem em policlínica	Superior completo	Estatutário	29 anos	Atualizado
8	Parda	Técnico de Enfermagem em USF	Superior completo	Estatutário	10 anos	Atualizado
9	Parda	Gestora	Superior completo	Estatutário	6 anos	Atualizado
10	Parda	Gestora	Superior completo	Contrato	3 anos	Atualizado
11	Parda	Gestora	Superior completo	Estatutário	22 anos	Desatualizado
12	Branca	Gestora	Superior completo	Estatutário	8 anos	Atualizado
13	Parda	Gestora	Superior completo	Estatutário	8 anos	Atualizado

ACS: agente comunitário de saúde; CLT: *Consolidação das Leis do Trabalho*; SUS: Sistema Único de Saúde; USF: unidade de saúde da família.

Fonte: elaboração própria.

As entrevistas foram gravadas e transcritas, o percurso da coleta foi iniciado com uma gestora da equipe do PMI, em seguida foram entrevistados usuários e profissionais de saúde, de modo alternado, obedecendo as agendas de ida ao campo, e por fim, foram realizadas as entrevistas com as demais gestoras. Um diário de campo foi produzido a partir desse trânsito, ampliando as possibilidades de registro como áudios, notas, reflexões, apontamentos e descrições do percurso para condução da cartografia ^7^.

A pergunta disparadora para a entrevista versou sobre o acesso ao imunobiológico e a decisão de iniciar e não continuar com as doses ofertadas até 2023. Para as profissionais de saúde e gestoras, a pergunta disparadora foi realizada considerando a população acima de 18 anos e a própria experiência de vacinação. Perfis foram identificados para os entrevistados usuários, após essa etapa, a leitura dos dados foi retomada com referência ao quadro de análise baseado na TAR ^7^ (Quadro 2).

**Quadro 2:** Quadro referência da Teoria Ator-Rede (TAR) ^7^ para análise das entrevistas realizadas com pessoas com esquema vacinal incompleto para a COVID-19. Pernambuco, Brasil, 2024.

QUADRO DE ANÁLISE DAS ENTREVISTAS	
Contexto	Compreende a descrição da situação pelo entrevistado ou diário de campo em que se identifica o movimento de agenciamento entre os atores humanos e não-humanos da situação descrita
Agenciamento e os atores	O movimento de agenciamento entre os atores humanos e não-humanos da situação descrita
Porta-vozes	Quem/que expressou o que os atores comunicam
Inscrição	Codificação das ações, que permite dar materialidade ao fenômeno, ou seja, transportá-lo, reconhecê-lo

Fonte: elaboração própria.

Na leitura das transcrições e diário de campo foram destacados o contexto, agenciamentos e seus atores, porta-vozes e dispositivos de inscrição, organizados em quadros com o suporte do Microsoft Excel (https://products.office.com/) e do software ATLAS.ti 24 (http://atlasti.com/). Após a organização, aproximações temáticas foram realizadas ancoradas aos conteúdos destacados no contexto e agenciamento, sinalizado no Quadro 2. Desse modo, agrupamentos temáticos (AT) foram construídos para subsidiar o mapeamento das controvérsias.

O Quadro 3 expõe os AT identificados por fonte de produção de dados, os atores/actantes e as controvérsias mapeadas. As dinâmicas das associações híbridas entre os atores humanos e não-humanos foram fundamentais para a análise, uma vez que, ainda que os atores se repetissem entre os AT, suas performances se deram de modo distinto. A depender de cada agrupamento, os mesmos atores que transformaram as interações em determinadas redes (os mediadores), em outras conexões, transportaram significados sem modificá-los ^7^. Observou-se que as redes sociotécnicas emergiram nos rastros das interações entre os atores mediadores ^7^, ou seja, actantes (humanos e não-humanos) que agenciaram as conexões acerca da hesitação vacinal.

**Quadro 3:** Quadro síntese das controvérsias mapeadas a partir das relações entre os agrupamentos temáticos. Pernambuco, Brasil, 2024.

FONTE	AGRUPAMENTOS TEMÁTICOS	ATORES/ACTANTES	CONTROVÉRSIAS
Usuários	AT1: As experiências pessoais e coletivas de confiança e desconfiança na vacina da COVID-19 AT3: A vacinação como ação coletiva *versus* a vacinação como ação individual AT5: Vacina e ideologia política AT6: Vacina, informação e desinformação	Humanos: Profissional de saúde Pessoas vacinadas Ex-presidente Jair Bolsonaro Pessoas hesitantes Pessoas antivacinação Familiares Profissionais vacinadores Divulgadores científicos (Luana Araújo; Átila Iamarino) Não humanos: Vacina da COVID-19 Sintomas pós-vacina - reações adversas à vacina da COVID-19 Campanhas de vacinação Passaporte vacinal (normativas) - Auxílio Brasil Passaporte vacinal (normativas) - Trabalho Redes e mídias sociais Unidades de saúde Movimento antivacina Notícias falsas Dados de mortalidade por COVID-19 Calendário vacinal Notas técnicas e normativas sobre vacinação Informação sobre a vacina Ideologia político-partidária	C1: A disputa pela confiança
Profissionais e gestores de saúde	AT1: Motivadores para descontinuidade do esquema vacinal da COVID-19 AT4: As consequências da desconfiança da vacina da COVID-19		
Diário de campo	AT2: A relação confiança e desconfiança na vacina da COVID-19 AT3: Motivadores e desmotivação à vacinação da COVID-19 AT5: Consequências da pandemia da COVID-19 para vacinação		
Usuários	AT1: As experiências pessoais e coletivas de confiança e desconfiança na vacina da COVID-19 AT2: O medo como elemento da hesitação e recusa à vacina AT5: Vacina e ideologia política AT6: Vacina, informação e desinformação	Humanos: Profissional de saúde Pessoas vacinadas Pessoas próximas (amigos e familiares) Ex-presidente Jair Bolsonaro Pessoas hesitantes e antivacinação Representantes religiosos Não humanos: Vacina da COVID-19 Campanha de vacinação Experiência pessoal Sintomas pós-vacina - reações adversas à vacina da COVID-19 Medo Desinformação Redes e mídias sociais Religião - misticismo - teorias Movimento antivacina Notícias falsas Dados de mortalidade por COVID-19 Ideologia político-partidária	C2: A disputa pela informação
Profissionais e gestores de saúde	AT1: Motivadores para descontinuidade do esquema vacinal da COVID-19		
Diário de campo	AT4: Vacina, crença e percepção		
Usuários	AT4: O acesso à vacina e a manutenção do esquema vacinal AT6: Vacina, informação e desinformação	Humanos: Pessoas com esquema incompleto Profissionais de saúde Gestores de saúde Profissional vacinador Não humanos: Vacina da COVID-19 Aplicativo para o agendamento e internet Unidades e salas de vacinação Rotina pessoal e dos serviços de vacinação Gestão municipal Estratégias intersetoriais Entes federativos Energia elétrica Frascos multidoses Comprovante de residência Informação	C3: A disputa diante das barreiras de acesso e necessidade de ampliação da cobertura vacinal
Profissionais e gestores de saúde	AT2: Estratégias para adesão e acesso vacinal da COVID-19 AT3: Barreiras de acesso para a vacinação contra COVID-19		
Diário de campo	AT1: Elementos sobre o acesso à vacinação da COVID-19		

Fonte: elaboração própria.

As controvérsias (desafios e conflitos) foram mapeadas a partir dessas interações, produtos do hibridismo que se conformam de modo plural, temporário e oscilante. Nesse sentido, não se pretendeu resolver os conflitos e fragilidades, mas rastrear as concessões, disputas, atores e agenciamentos presentes ^7^. Assim, a descrição dessas relações conduziu a apresentação dos resultados por controvérsia. A pesquisa obedeceu aos trâmites éticos exigidos pelo Comitê de Ética do Instituto Aggeu Magalhães, Fundação Oswaldo Cruz (CAAE: 73386523.2.0000.5190).

## Resultados

Vinte e um usuários relataram a não intenção de prosseguir com o esquema vacinal e 15 com intenção. Referente à caracterização das pessoas entrevistadas, a média de idade foi de 45,53 anos, pouco mais da metade se identificou com o gênero feminino, 86,1% das pessoas entrevistadas foram negras (pardas e pretas), grande parte identificou-se como autônoma ou desempregada. Cerca de 94,4% das pessoas tinham a renda familiar mensal inferior a dois salários mínimos, apenas uma pessoa entrevistada possuía plano de saúde. Cerca de 63,88% das pessoas seguiam a religião evangélica. Foram identificados três perfis de usuários: confiante na primeira dose da vacina (UC), hesitante na primeira dose da vacina (UH) e vacinados apenas por exigência (UVE). As informações sobre o perfil detalhado dos usuários entrevistados estão na Tabela 1.

**Tabela 1:** Perfil das pessoas entrevistadas com esquema vacinal incompleto para a vacina contra a COVID-19, por intenção de seguir com a vacinação contra a COVID-19. Pernambuco, Brasil, 2024.

Variáveis	Com intenção	Sem intenção	Total
Início da campanha de vacinação			
Confiantes	13	8	21
Hesitantes	1	7	8
Vacinados por exigência	1	6	7
Dose da vacina no momento da entrevista			
Dose 3	9	9	18
Dose 2	5	9	14
Dose 1	1	3	4
Gênero			
Feminino	7	13	20
Masculino	8	8	16
Raça/Cor			
Pardo	11	12	23
Negro	3	5	8
Branco	1	4	5
PCD			
Não	14	20	34
Sim	1	1	2
Renda familiar (salários mínimos)			
Até 2	15	19	34
2-5	0	2	2
Escolaridade			
Médio completo	10	9	19
Fundamental completo	3	2	5
5º ano incompleto	0	4	4
Superior completo	0	3	3
Superior incompleto	2	1	3
5º ano completo	0	2	2
Fez parte da população prioritária para vacinação			
Não	14	17	31
Sim	1	4	5
Tipo de vínculo trabalhista			
Autônomo	9	7	16
Desempregado	4	10	14
CLT	2	2	4
Outro	0	2	2
Possui plano de saúde			
Não	14	21	35
Sim	1	0	1
Religião			
Evangélico	6	17	23
Católico	5	3	8
Sem religião	4	0	4
Espírita	0	1	1

CLT: *Consolidação das Leis do Trabalho*; PCD: pessoa com deficiência.

Fonte: elaboração própria.

Os AT identificados a partir dos dados produzidos subsidiaram o mapeamento das seguintes controvérsias: a disputa acerca da confiança no cenário da vacinação da COVID-19, a disputa sobre o debate da informação, e a terceira atrelada as disputas da ampliação da cobertura vacinal diante das barreiras de acesso (Quadro 3).

### A disputa pela confiança

Confiar no imunobiológico não foi suficiente para prosseguir com a intenção na manutenção do esquema vacinal da COVID-19. No centro da construção do sentimento de confiança e sua manutenção, esteve a ideia da imunização como ação de proteção coletiva, sobressaindo a escolha individual, e tal construção esteve permeada por narrativas de experiências pessoais na pandemia, como a perda de entes queridos. Para parte das pessoas confiantes, acompanhar a vacinação das pessoas próximas, as campanhas e a compreensão da vacina como uma ação coletiva foram fundamentais para adesão, como sinalizado no trecho 1 (Quadro 4).

**Quadro 4:** Trechos retirados dos dados produzidos para a pesquisa. Pernambuco, Brasil, 2024.

CONTROVÉRSIA	TRECHOS DESTACADOS
C1: A disputa pela confiança	Trecho 1: “*Eu esperei ansiosa para chegar a minha vez de tomar a vacina. Eu vi a necessidade e quando começou a vacinar os primeiros grupos já melhorou. A pandemia já foi diminuindo* (...) *só lamento não ter vindo antes* (...) *inclusive perdemos um irmão, logo no início. É, se tivesse chegado antes, talvez poderia ter salvado mais pessoas, porque demorou um pouquinho*” (UC)
	Trecho 2: “*Eu não tomei* [dose de reforço]*, porque assim no meu ponto de vista, para minha pessoa, eu achei até agora que não é necessário, entendeu? Mas eu não sou contra* [a vacina da COVID-19]*, eu sou a favor. Porque eu presencie muita gente morrendo no hospital, nas UPAs*” (UH)
	Trecho 3: “*Porque tinha que tomar que o povo ficava dizendo coisa, ficava dizendo que tinha que tomar por conta da Bolsa Família, tinha que tomar por conta da coisa que a gente fosse receber, resolver em algum canto. Aí tinha que procurar, porque todo canto que chega era ‘cadê o cartão da vacina?’ Tinha que mostrar*” (UH)
	Trecho 4: “*Aí, Bolsonaro tinha falado que não ia, que os patrões não podiam fazer isso, botar as pessoas para fora por causa da vacina, porque a vacina estava a critério das pessoas, se a pessoa quisesse tomar ou não, ele mesmo disse: eu mesmo não vou tomar. E parece que a mãe dele não tomou*” (UVE)
	Trecho 5: “*Eu acho que foi relativo, assim as questões políticas mesmo da época da campanha nacional antivacina, por parte da própria Presidência da República, o presidente naquele momento e essa influência política nas questões religiosas, que fizeram também com que as pessoas de determinadas religiões não quisessem por conta dessas questões políticas. O que eu via mais era isso. Até porque, veja, é vacina nenhuma, até hoje eu trabalho com vacina desde 95. Há quase 30 anos que eu trabalho com vacina e ninguém nunca procurou saber como foi que foi feita uma vacina, quais foram as pesquisas, isso nunca existiu. Esse tumulto todo só existiu agora*” (P7)
	Trecho 6: “*Uma técnica de enfermagem, ela disse isso ‘não é aquela vacina que está vencida’, eu digo ‘não é aquela vacina que está vencida, porque a gente não traz vacina que está vencida, a gente está com vacina que está com validade estendida’. E aí eu fui conversar com ela, porque assim, você não pode dizer isso na frente do paciente, porque a gente já estava vivendo que a pessoa já não acredita. E eu ainda chegar na frente do paciente, cadê a sua ética profissional?*” (G2)
C2: A disputa pela informação	Trecho 7: Entrevistada: “*Eu acredito que a vacina não é feita e em poucos dias. É descobrir o mistério para detectar essa doença, a COVID-19. Por isso que eu não acredito nessa vacina, foi um teste, e fizeram todos de cobaia, isso que eu acredito. Que não seja o que eu penso, né? Que é para destruir a humanidade em vez de ajudar a se recuperar dessa doença*” Entrevistadora: “*A senhora acha que é para destruir, como assim?*” Entrevistada: “*Futuramente vem os efeitos. Como muitos estão infartando. Muitos estão tendo, é como tipo, é sobre o infarto, mesmo e outras doenças que estão aparecendo*” (UVE)
	Trecho 8: “*Eu não tomei as outras doses porque para mim é uma vacina experimental, uma vacina que foi feita, eu acho que em um ano, para mim uma vacina dessa é experimental aí, por isso que eu não quis tomar outras doses*” (UVE)
	Trecho 9: “*Hoje em dia a gente vê mais gente com medo. E assim, de certa forma é houve, porque a AstraZeneca foi suspensa. Então houve realmente notícias que até então ditas como fake-news, mas não eram, porque hoje foi comprovada e ela foi retirada do mercado. Então eu acho que é por conta de como é uma doença nova. É algo novo, né? E aí eu acho que ainda vem muitos estudos por aí. E aí em países, a vacina para as crianças não acontece. Elas foram banidas realmente, né?*” (G4)
	Trecho 10: “*É fiquei assim, dúvida, né? Entre sim e não. Pelas informações negativas de um e de outro, de que pessoas estavam se sentindo mal, passando mal, tendo problema, certo problema de pulmão, essas coisas, então eu parei. Foram informação de terceiros, por passarem mal entre família, parentes e acharam que era devido a vacina*” (UC)
	Trecho 11: “*De lá para cá, fiquei todo dia com dores na minha cabeça, que eu não tinha isso, depois que eu tomei a vacina foi que eu fiquei com essa sequela de dor de cabeça, vou dormir bem e me acordo com a cabeça cheia, aquela dorzinha chata. E assim, todo dia dói mesmo. Foi depois que eu comecei tomar essa vacina*” (UH) [após finalizar a gravação da entrevista, o entrevistado relatou que ele e a esposa tiveram sintomas gripais característicos da COVID-19, como anosmia]
	Trecho 12: “*Depois da segunda dose, eu fiquei com muito medo, porque muitas pessoas disseram que era para matar, que era para infartar. Não tomo mais nenhuma e aí não tomei mais não e nem vou tomar... a gente ia para posto de saúde e o pessoal ‘não toma não, que vai matar a gente’*” (UH)
	Trecho 13: “*Encontrei um senhor aqui na porta da unidade, disse que tomou as 4 doses e não toma mais. Ele disse que não tomava mais, porque é uma vacina para matar os velhos, que viu uma entrevista. Os Illuminati, que as três famílias mais ricas do mundo dizendo que queria acabar com os velhos, pois tem muita gente no mundo, e que criam algumas coisas para matar essas pessoas e diminuir a quantidade, o gasto com a previdência, para não pagar a aposentadoria. Que agora está um rolo, que a saúde fica obrigando todo mundo a tomar, principalmente os velhos, atrelando ao benefício. Para ele era como se a vacinação fosse uma conspiração*” (trecho do diário de campo)
	Trecho 14: “*Eu acho que é a própria besta-fera que está fazendo isso para matar o povo, para diminuir a população, porque realmente tem muita gente, aí eles inventaram uma forma de enganar e dizer que vai curar e não cura ninguém, e vai matar*” (UH)
	Trecho 15: Entrevistada: “[O vídeo] *foi sobre a política. Que a tendência dos cabeças, os que estão de frente* [referência ao atual governo Lula]*, é para tirar as leis do Brasil e comandar como eles querem. Porque a gente já está quase sem lei mesmo, os bandidos não vão preso, mais mata, rouba, é preso em um dia no outro já está difícil na rua, combater, fazendo tudo de novo*” Entrevistadora: “*E a senhora acha que tem alguma coisa a ver com a vacina, com a obrigatoriedade?*” Entrevistada: “*Tudo, a vacina junto com a bandidagem tem tudo a ver com a política. Com quem está como cabeça de frente. Comandante tem tudo a ver*” (UVE)
	Trecho 16: “*Eu passei uns dias pensando se eu deveria tomar ou não, porque aconteceu muitas coisas, parente com gente mais próxima, amigo, aí eu perdi muitas amigas minha depois que tomou a terceira dose, tinha saúde, não tinha problema nenhum e tomou a terceira dose e sentiu mal, foi socorrido e faleceu. Aí eu fiquei com medo de tomar. Fiquei pensando, se tomar, se deveria tomar ou não. Aí depois eu achei que deveria tomar pelo menos a primeira dose. Aí só foi o que eu fiz, tomei a primeira dose. Meus filhos, graças a Deus, tudo, minha filha tomou todas as doses, tudo direitinho, ela e meu genro. Eles pegaram a primeira vez e agora, pela segunda vez de novo, pegaram novamente e foi mais fraca por conta da vacina que eles tomaram. Não foi tão forte como logo do início da pandemia*” (UH)
	Trecho 17: Entrevistada: “*A maioria se vacinou, já teve outros que não quiseram*” Entrevistadora: “*E tu sabe quais foram os motivos que essas pessoas*” Entrevistada: “*Bolsonaristas*” (UC)
	Trecho 18: *“Porque como Bolsonaro disse, que a vacina para ser lançada tinha que passar por vários processos. E ela foi muito rápida, entendeu? Se não teve, a gente foi a cobaia. A gente foi cobaia dessa vacina, muita gente que faleceu, uma colega minha mesmo faleceu. Porque eles não procuram saber. Assim, porque as vezes a gente está com problemas de saúde e a gente não sabe. Aí tomar vacina para uma coisa e às vezes prejudica o outro*” (UVE)
C3: A disputa diante das barreiras de acesso e necessidade de ampliação da cobertura vacinal	Trecho 19: “*A primeira dose foi fácil, eu tomei porque eu ia trabalhar na firma e eu tinha que tomar, e a segunda, toda vez que eu passava no posto, não tinha, e eu estava tendo de fazer um serviço, aí na data que estava disponível, eu não podia ir*” (UC)
	Trecho 20: “[as primeiras doses] *eu me vacinei no shopping, agendei pela internet, me vacinei lá e depois tomei aqui* [unidade de saúde]*. Foi fácil, fui de ônibus. Mas eu nem sabia que tinha* [dose de reforço]” (UC)
	Trecho 21: “*Eu acho importante a vacinação, o que de fato não entendo é a questão desse reforço. Na nossa, na minha cabeça, precisamente, quando se foi divulgado a vacina era 1 dose, ou 2, só que eu sou leiga mesmo desse assunto, não procuro mais entender essa história. É que, teoricamente, é o que eu sei, parece que sempre a gente vai ter que tomar o reforço. Então eu ainda não tenho essa formulação de decisão na minha mente com relação a isso*” (UC)

G: gestora; P: profissional de saúde; UC: usuário confiante; UH: usuário hesitante; UPA: unidade de pronto-atendimento; UVE: usuário vacinado por exigência.

Fonte: elaboração própria.

Os acolhimentos na sala de imunização ou na rotina do serviço de saúde facilitaram a adesão e o sentimento de confiança no imunobiológico. O relato da experiência pessoal e de familiares dos profissionais de saúde, a informação sobre os riscos da COVID-19, a mudança de cenário na pandemia pós-vacinação e a comparação com outras vacinas durante a vida do usuário incentivaram a decisão, apresentando o profissional de saúde como principal porta-voz para a adesão.

A dimensão da construção de um sentimento de confiança esteve atravessada por profissionais vacinadores e não vacinadores. Para os profissionais foi importante o auxílio de fontes de informação, como divulgadores científicos popularizados pela Comissão Parlamentar de Inquérito da COVID-19, no confronto às notícias falsas, especialmente para os não vacinadores, que assumiram uma postura responsável e coletiva de cuidado frente ao contexto de pouca formação sobre o imunobiológico para esse perfil de profissional.

Mesmo com uma compreensão positiva da vacinação, a mudança no cenário pandêmico e o afrouxamento das medidas não farmacológicas, como a desobrigação das máscaras e a retomada das aglomerações, surgiram como motivadores para a descontinuidade da vacina, apesar das indicações de manutenção do esquema pelas autoridades sanitárias.

Entre os confiantes, a perspectiva da imunização fundada na individualidade, foi definidor para não prosseguir com a vacinação, exemplificado na defesa de ao menos uma dose ser suficiente para não adoecer, na confiança no próprio sistema imunológico ou em medidas individuais de proteção com o foco na contaminação individual, desvencilhado da proteção coletiva, exemplificado no trecho 2 (Quadro 4).

O debate sobre a obrigatoriedade atrelado ao passaporte vacinal para o acesso aos serviços públicos, manutenção do emprego ou aos benefícios de distribuição de renda, como percebido do trecho 3 (Quadro 4), gerou desacordos entre usuários dos serviços, reafirmando o conflito entre a vacinação como ação individual ou coletiva, reacendido na dimensão do discurso da liberdade individual.

No período da campanha do imunizante, o Estado de Pernambuco publicou o *Decreto nº 51.864/2021* ^20^, sobre a exigência do passaporte vacinal para permanência e acesso aos órgãos e entidades públicas de administração direta e indireta. Já a *Portaria nº 597/22* do Ministério da Saúde ^21^, sinaliza o comprovante vacinal como equipamento de proteção individual para o trabalho, o que pode ter justificado a exigência dos empregadores.

Sob a gestão do ex-presidente Jair Bolsonaro, a vacinação da COVID-19 esteve desvinculada do benefício de transferência de renda, na época nomeado de Auxílio Brasil (*Medida Provisória nº 1.061/2021*) ^22^. Para os profissionais e gestores, o período de desvinculação do Auxílio Brasil foi negativo para a adesão ao imunizante, especialmente para a recusa, em um processo de expansão de desconfiança. O resgate posterior na relação benefício e vacinação, bem como a relação passaporte vacinal e emprego, se configurou como importante ator para a adesão, mesmo diante da desconfiança.

O desacordo com a exigência desse passaporte esteve associado a defesa da liberdade individual de escolha. Notou-se que a compreensão do comportamento individual da vacinação esteve acompanhada do desincentivo à vacina da COVID-19, que teve como principal porta-voz o ex-presidente Jair Bolsonaro, como apontado no recorte do trecho 4 (Quadro 4).

O ex-presidente foi agenciador para a ampliação da desconfiança vacinal, sendo sugerido como principal porta-voz de uma campanha antivacinação, mediada por discursos religiosos, ideológicos e pela disseminação da desinformação e notícias falsas, por meio das redes e mídias digitais (Instagram, YouTube, WhatsApp) sobre o imunizante e possíveis sequelas, como a morte. No trecho 5 (Quadro 4), a profissional entrevistada destacou essa interação ao referir-se ao cenário investigado.

Outros motivadores estiveram presentes para as pessoas não prosseguirem com o esquema vacinal, compondo assim uma rede sociotécnica da desconfiança. A dinamicidade das notas técnicas e mudanças nos esquemas, a variedade de laboratórios e as modificações referentes ao manejo das doses, as reações adversas, os profissionais de saúde que desestimularam a adesão, sob a justificativa da autoridade sobre o assunto, fragilizaram o sentimento de confiança na vacina da COVID-19. Mesmo para as pessoas confiantes, essa dinamicidade fortaleceu a desconfiança. No trecho 6 (Quadro 4), a gestora entrevistada relata uma situação que ocorreu em seu local de trabalho.

Os profissionais vacinadores vivenciaram situações de desgaste na sala de vacina na lida com o novo ator: a hesitação vacinal. Os indícios da pouca ou nenhuma confiança no imunizante foram percebidas nas pessoas hesitantes ou vacinadas por exigência, e a sala de vacinação desenhou-se como espaço de conflito pessoal, com dúvidas e questionamentos sobre qual ator/porta-voz confiar. Nesse sentido, desafios estão colocados no cenário de atraso vacinal em relação ao esquema da COVID-19, assim como para o PNI, especialmente diante da manutenção e atualização do calendário vacinal, da incrementação de novos imunobiológicos e, diretamente, para a lida rotineira do profissional/equipe vacinadora.

### A disputa pela informação

Notícias falsas e boatos foram apresentados com características distintas na rede sociotécnica acerca da disputa pela informação, mesmo contendo conteúdos similares. Em alguns contextos, as notícias falsas ou *fake news* surgiram nos relatos no campo da certeza de quem anunciou e definiu o conteúdo como falso. Em outros cenários, o mesmo conteúdo das notícias falsas foi parâmetro para a certeza, lida na experiência como uma informação verdadeira, como demonstrados nos trechos 7 e 8 (Quadro 4).

Os materiais da campanha da vacinação ou vídeos e mensagens nas redes sociais foram inscrições destaques nas duas perspectivas. Entre profissionais/gestoras, com unanimidade no discurso de incentivo à imunização, notícias falsas também surgiram para embasar o relato sobre o imunizante, como no trecho 9 (Quadro 4). Especificamente, sobre a vacina AstraZeneca e a suspensão da vacinação infantil da COVID-19 foram identificadas como notícias falsas pelo Ministério da Saúde. 

Diferente das notícias falsas, os boatos surgiram como um conteúdo oral atrelado à experiência pessoal de pessoas conhecidas e grupos. O boato foi apresentado com o conteúdo similar ao da notícia falsa, no entanto, esteve acompanhado da ponderação em crer ou não. O relato do outro e a experiência pessoal estiveram no centro da tomada de decisão, como demostrado no trecho 10 (Quadro 4).

Na dimensão da experiência, os relatos das sequelas pós-vacinação ganharam visibilidade, carregaram o *status* da verdade, da experiência real de relacionar essas sequelas ao imunobiológico. Mesmo em situações com episódios de sintomas gripais característicos da COVID-19, a infecção não esteve vinculada aos sintomas/sequelas relatadas, mas ao imunizante, como sinalizado no trecho 11, acompanhado de uma nota do diário de campo (Quadro 4).

A notícia falsa e o, aqui denominado, boato integraram o campo da desinformação, com os seguintes conteúdos: “*a vacina é experimental pois não teve de 5 a 10 anos de pesquisa*”, “*ela não tem evidência*”, “*a população foi cobaia*”, “*a vacina deixa sequelas como problema de pulmão, trombose, infarto*”, “*a vacina mata e isso pode ocorrer em cinco anos*”, “*vacinas vencidas foram distribuídas*”, “*o vírus foi criado em laboratório*”, “*a intubação, não o vírus, foi o responsável pelas mortes*”, “*hospitais forjaram dados sobre o internamento por COVID-19*”.

Os conteúdos acima estiveram associados ao medo, com inscrições nas mídias e redes sociais (Instagram, WhatsApp). O medo, na dimensão do boato esteve vinculado à informação da morte e sequelas, sempre com uma referência próxima familiar ou de grupos de trabalho, igreja, vizinhança, como exemplificado no trecho 12 (Quadro 4).

O medo foi central e agenciador na experiência dos hesitantes, mas também entre os confiantes. Como exemplo, a emoção esteve presente na reação adversa vivida em doses anteriores, inclusive entre profissionais, no receio a dor causada pela injeção ou relacionado à disseminação de experiências ruins com a vacinação da COVID-19.

Teorias conspiratórias, personagens místicos ou porta-vozes dos conteúdos antivacina tomaram uma dimensão maior que a experiência vivida, como o fato de se vacinar ou na mudança do cenário epidemiológico, após início da campanha. Exemplificado nos trechos 13, 14 e 15 (Quadro 4), houve a crença em um experimento global acerca da vacinação contra a COVID-19, com teorias sobre morte de pessoas idosas para diminuir custos com a aposentadoria e sobre a morte das pessoas mais pobres; nessas perspectivas, a vacina da COVID-19 seria um arranjo conspiratório.

Houve o registro de orientações religiosas direcionando para não adesão, além da crença em aspectos místicos e religiosas, como figuras malignas, que, via imunizante, agiriam para matar. A crença da vacinação como conspiração político-ideológica e criminalidade também foi sinalizada. Baseado em notícia falsas, os boatos foram apresentados nas entrevistas alimentando aspectos negativos, como a incerteza sobre o benefício da vacina, mesmo diante dos indícios cotidianos da sua eficácia, como sinalizado no trecho 16 (Quadro 4).

A informação assumida como falsa esteve atrelada aos grupos que minimizaram ou negaram a gravidade da pandemia, como grupos antivacina. Neste panorama, o uso de celular, redes e mídias sociais, profissionais de saúde e o ex-presidente Jair Bolsonaro foram atores dessa rede sociotécnica (ver trechos 17 e 18; Quadro 4).

O debate ideológico foi mediador, vinculados às notícias falsas e aos boatos. Parte das pessoas entrevistadas fizeram críticas ao cenário político-ideológico dos primeiros anos de imunização da COVID-19, assim como outros demonstraram o apoio. Para alguns entrevistados, citar o nome de representações político-ideológicas poderia deslocar o conteúdo da entrevista para não neutralidade; ou seja, ser imparcial significou fazer a leitura da vacinação da COVID-19 sem citar porta-vozes políticos.

Nesse sentido, especialmente para alguns profissionais e gestores, a ideologia política poderia ser um ator desqualificador do debate. Para algumas entrevistadas, mesmo ao expor atores considerados importantes para a não adesão vacinal ou para expansão da hesitação vacinal no Brasil, o cenário político-ideológico do país não foi um ator importante nesta rede.

### A disputa barreiras de acesso e necessidade de ampliação da cobertura vacinal

O município se organizou na oferta do imunobiológico, inicialmente com salas de imunização e *drive-thru*, alguns improvisados em dispositivos sociais (*shopping*, supermercados, igrejas entre outros), com o agendamento online, via site específico, e com a exigência de comprovante de residência para o acesso ao imunizante. A descentralização da vacina ocorreu à medida que foi ampliando a sua disponibilidade nas unidades de saúde, sem necessidade de agendamento online ou comprovante de residência. Neste período, a busca pelo imunizante retornou à universalidade do acesso, com a organização dos usuários nas unidades de saúde, a partir de fichas para filas por ordem de chegada.

Foi referido o acesso facilitado pelo uso do aplicativo/site do município para agendamento, com o suporte da unidade de saúde e outras estratégias, como a *Van da Vacinação*, automóvel equipado com o imunobiológico e equipe de saúde, especialmente para áreas com frágil cobertura de atenção básica; equipe domiciliar exclusiva para vacinação de idosos e acamados; ações intersetoriais com outras Secretarias Municipais, articulações com representações comunitárias.

Abordagens no nível da gestão foram indicados para ampliar o acesso às pessoas em atraso vacinal, tais ações garantindo o reabastecimento das unidades a partir do planejamento da demanda local, atividades no território como microplanejamento, mapeamento, monitoramento e busca-ativa de determinados grupos, ações de educação em saúde e formação e qualificação da equipe vacinadora.

Mesmo diante das estratégias, a existência de barreiras foi sinalizada. A exigência do comprovante de residência configurou-se como uma barreira para as primeiras doses. Uma entrevistada, por exemplo, precisou retornar ao município de nascimento para iniciar o esquema vacinal por falta do comprovante. Gestoras e profissionais apontaram que a exigência pode ter se configurado como barreira de acesso, que mesmo havendo flexibilidade de algumas equipes vacinadoras, pessoas não foram imunizadas por falta da comprovação, mesmo residentes no município.

A falta do imunobiológico nas unidades, aspectos da infraestrutura, como a falta de energia, a distância das unidades com características rurais dos locais de abastecimento, a falta de informação sobre agendamento da vacina, a dificuldade com a internet e o descompasso entre o serviço de saúde e a rotina de trabalho/atividades diárias, foram identificadas, como indicado no trecho 19 (Quadro 4). Para profissionais e gestores, a necessidade de organização diante dos frascos multidoses, as cotas vacinais por municípios e as dificuldades de abastecimento na relação entre os entes federativos foram barreiras para o acesso.

No âmbito da sala de vacina, destacou-se nas USF a sobrecarga de um mesmo técnico de enfermagem para as atividades de vacinação, entre outras atividades como as visitas domiciliares, realização de curativos, acolhimento, ações que não garantem a disponibilidade do serviço a partir da necessidade de quem vai em busca do imunobiológico. Somam ao cenário, a necessidade do registro no sistema online ou em ferramenta própria do processo de trabalho (livro de registro de vacinas), as dificuldades no manejo do sistema e a qualidade da internet, aspectos que tornam o trabalho mais desgastante para o profissional.

A fragilidade na comunicação governamental sobre o imunizante, com destaques para a compreensão sobre a vacina, a sua proteção coletiva, as doses de reforço, as reações adversas e as pesquisas científicas sobre o tema, além da desinformação entre confiantes, hesitantes e vacinados por exigência, foram elementos dificultadores para a manutenção do esquema, como sinalizados nos trechos 20 e 21 (Quadro 4).

Para as gestoras e profissionais, as questões da falta de informação, por exemplo, protagonizaram a dificuldade da adesão vinculado ao interesse do usuário, mas não como barreira de acesso. Foi comum a compreensão do acesso vinculada à disponibilidade do imunizante. Ou seja, prioritariamente o acesso atrelou-se ao abastecimento ou desabastecimento da unidade, somado ao interesse do usuário em prosseguir com a vacinação e a sua necessidade de se adaptar à disponibilidade da vacina nas unidades de saúde.

## Discussão

Os agenciamentos na rede sociotécnica acerca das controvérsias da hesitação vacinal direcionaram para a necessidade de compreensão das relações entre confiar e desconfiar no imunizante como uma característica fluída entre perfis confiantes, hesitantes e vacinados por exigência. Nesse sentido, a desconfiança esteve presente mesmo diante da adesão, cenário desafiador para a vacinação da COVID-19.

A confiança nas autoridades sanitárias, profissionais de saúde, formuladores de políticas é um eixo central na literatura acerca da hesitação vacinal ^1^ ^,^ ^4^ ^,^ ^5^ ^,^ ^6^. O sentimento passa pela disputa entre atores e porta-vozes pró-vacinação ou contra vacinação. Para o fortalecimento da confiança, a atuação do profissional de saúde surge como estratégia de informação, segurança e definidor para vinculação ao sentimento pró-imunização ^23^. Nesse cenário, cabe destacar a necessidade de integração, valorização e qualificação dos profissionais vacinadores e não vacinadores para o fortalecimento da crença e do acesso ao imunobiológico.

A expansão do ideal de responsabilidade coletiva vinculada a imunidade e proteção coletiva conformou como aspecto essencial ^5^. Essa dimensão, presente no modelo 5Cs ^5^, emergiu como mobilizadora para a continuação do esquema vacinal, sugerindo que quando não existente, a percepção da vacina como escolha individual justificaria o desinteresse pelo imunobiológico, mesmo em pessoas confiantes. A compreensão da vacinação como coletividade foi confrontada pela percepção da imunização como liberdade de escolha/direito individual nos três perfis.

O debate entre o direito individual e coletivo nesses contextos não foi inaugurado na pandemia da COVID-19, a vacinação compulsória segue esfera de interesse para o direito e a bioética, no qual, em cenário pandêmico, prioriza a necessidade do coletivo para proteção populacional ^24^. No Brasil, a liberdade e direitos individuais estiveram associados aos discursos do ex-presidente Jair Bolsonaro e sua relação com negacionistas, liberais econômicos, conservadores sociais, no bojo da politização da vacina ^9^ ^,^ ^12^.

O discurso individualista confrontou a Organização Mundial da Saúde (OMS), que disseminou a mensagem da confiança no imunizante, da transparência da informação e da imunização como responsabilidade e proteção de todos, no intuito de fragilizar a hesitação e suas consequências ^25^. A estratégia da responsabilização coletiva convidou as reflexões sobre as barreiras de acesso e a desigualdade vacinal, especialmente na relação com as iniquidades sociais estruturais.

Apesar da fragilidade dos dados sobre a relação desigualdade vacinal e marcadores sociais da desigualdade e diferença, estudos já apontam a desigualdade na vacinação da COVID-19, como por exemplo, a maior cobertura nos municípios com melhores indicadores socioeconômicos e maior população branca ^13^ ^,^ ^14^. No bojo da decisão em vacinar, autoras apontam a relação entre a perspectiva da individualidade, branquitude e o maior poder socioeconômico, reafirmando o caráter social e político da hesitação vacinal ^26^.

Foi identificado a percepção de que apenas a disponibilidade do imunobiológico na unidade garantiria acesso, e a responsabilidade individual como principal motivador/interesse para a adesão. Essa defesa se distancia da responsabilização coletiva e solidária, fortalece a perspectiva individualizante, contradiz o interesse e as estratégias de gestão descritas para a ampliação da cobertura do imunizante.

Barreiras de acesso foram apontadas e podem estar associadas, para confiantes e hesitantes, junto a mudança do cenário pandêmico, ao sentimento denominado de fadiga pandêmica e vacinal. Autoras identificaram, com adultos imunizados ao menos com duas ou três doses da vacina da COVID-19, o sentimento de fadiga ^10^, ou seja, de desmotivação/cansaço em relação às recomendações de saúde pública ^2^.

Nesse contexto, a informação foi definidora para esse cenário ^27^ ^,^ ^28^, o seu excesso e a dificuldade em atribuir veracidade à fonte assumiram interações com as barreiras de acesso, como dificuldade de distinguir informações corretas sobre a vacina da COVID-19. Na pandemia, a desinformação e o uso das tecnologias de informação e comunicação, no geral, estiveram vinculados à hesitação vacinal e ao impacto negativo das notícias falsas para a vacinação ^28^.

Os dados produzidos por esse estudo também corroboram com outras investigações ^29^ ^,^ ^30^ que apresentaram as dimensões das crenças, especialmente religiosas e teorias conspiratórias na percepção das consequências negativas da vacina. Nesse agenciamento, as emoções foram mediadoras. Emoções como o medo e a esperança integraram este cenário em uma dinâmica com as informações oriundas de fontes oficiais e não oficiais ^31^.

A esperança assumiu uma relação com a confiança na vacina e o medo interagiu com a desinformação e as experiências pessoais. O medo foi mediador para a ameaça diante dos imunizantes ^23^, configurando-se uma barreira afetiva para a vacinação ^31^. Essa emoção, que esteve indissociável da disseminação das notícias falsas em mídias, redes sociais e nas experiências pessoais, se destacou como ameaçadora, inclusive, para os confiantes na vacina.

As experiências sociais estiveram entrelaçado às respostas cognitivas e emocionais à vacinação, como identificado na literatura ao domínio pensar e sentir ^1^; autores sinalizaram que tais aspectos estão vinculados à aceitação vacinal ^32^. Diante desse debate, a complacência ^4^, compreendida como a ideia do risco associado à vacina ser maior que a infecção, em alguns cenários, inviabilizou a percepção de evidências positivas com a vacinação vivida no cotidiano, especialmente das pessoas hesitantes, inclusive se estendendo para outros imunobiológicos.

A rede sociotécnica ao redor da imunização remeteu a manutenção e evolução das interações, e envolveu vieses de omissão, confirmação e disponibilidade ^3^ ^,^ ^33^. Nos achados, o destaque ao viés de confirmação, ou seja, a tendência a usar as informações para confirmar sua experiência e crenças ^33^, na pandemia da COVID-19, somou-se as notícias falsas como fonte, tendenciando o viés de disponibilidade, crendo em uma consequência vacinal, desconsiderando os sintomas como uma consequência da infecção pelo SARS-CoV-2, por exemplo.

Foi comum remeter à vacina alguns sintomas, mesmo diante do histórico de infecção pelo SARS-CoV-2 e a possibilidade, por exemplo, da COVID longa. Apesar da condição ser mais frequente na população não vacinada, há indícios da condição em pessoas imunizadas, e ainda há dificuldade do sistema público de saúde em acolher essa demanda ^34^. Esse cenário nos leva a refletir sobre a consequência da falta da informação operar na atribuição de sintomas pós-vacinação, maquiando possíveis experiências de COVID longa.

A segunda controvérsia transitou pela representação do que se assume como verdade, da sua fonte e das emoções. A conformação das verdades esteve ancorada em proposições ^35^ que sustentaram essas redes sociotécnicas e seus efeitos, sejam proposições científicas que confrontaram as notícias falsas, sejam as proposições que performaram a desinformação ^35^. Ou seja, confiantes, hesitantes e vacinados por exigência, que desejaram ou não continuar com o esquema vacinal, interagiram com diferentes redes que sustentaram suas ações e garantiram o *status* de verdade.

Os achados destacaram a intensa mobilização do principal porta-voz do boicote à vacinação e a disseminação de notícias falsas, o ex-presidente Jair Bolsonaro, que tem sua desastrosa atuação analisada pela literatura, inclusive no aprofundamento de barreiras de acesso, como na operacionalização do PNI ^12^. Sabe-se que a implementação da vacina (aquisição, entrega, rede de refrigeração, manuseio, diversidade de insumos entre outros atores) impactou na aceitação vacinal ^36^.

Souto et al. ^23^ pontuam o ineditismo na história do PNI, em que órgãos públicos e coordenação federal foram determinantes para o desincentivo à adesão. Apesar do distanciamento entre imunização e tensionamento político surgir como tendência para alguns atores dessa investigação, é factível que no cenário brasileiro as disputas político-ideológicas emergiram como ator mediador na rede acerca da hesitação vacinal. A gestão de Jair Bolsonaro direcionou o percurso da vacinação da COVID-19 no Brasil ^9^, tensionou e agenciou controvérsias no campo da hesitação em um panorama marcado, também, pelas desigualdades vacinais ^13^ ^,^ ^14^.

## Considerações finais

A descontinuidade vacinal foi cenário para identificar as disputas em meio aos agenciamentos entre atores, porta-vozes, dispositivos de inscrição e mediadores de uma rede sociotécnica da hesitação vacinal. Os resultados reforçaram desafios para o cenário da imunização de pessoas com o esquema incompleto, sejam confiantes, hesitantes ou vacinados por exigência.

Destacaram-se as disputas pela confiança nos atores prós e contra vacinação, os conflitos entre fontes de informação expressos na fidelidade à própria experiência mediada pela desinformação, e o conflito para as políticas de vacinação, gestores e profissionais de saúde no descompasso entre barreiras de acesso e a necessidade de ampliação da cobertura vacinal.

O estudo apresenta limitações metodológicas, por retratar um cenário específico, em um determinado recorte de tempo e perfil de entrevistados. No entanto, os resultados convidam ao aprofundamento da problemática no campo da saúde pública, com outros desenhos metodológicos para: (a) indicar especificidades no diverso campo da hesitação vacinal; (b) investigar como o fenômeno se expressa em diferentes territórios e populações; e (c) fomentar contribuições para operacionalizar estratégias de ampliação da cobertura vacinal no cenário da imunização de pessoas com esquema incompleto.

O debate proposto reafirma abordar a hesitação vacinal compreendendo que a adesão não se configura sinônimo de aceitação. Pessoas, por diversos motivadores, podem aderir à vacinação e ainda assim permanecer hesitantes. Este estudo, apesar de localizar a pesquisa e descrever o perfil das pessoas entrevistadas, apontando aspectos como raça, classe, gênero e outros marcadores, não explorou o debate na relação com a hesitação e a descontinuidade vacinal. Nesse sentido, cabe destacar a importância do aprofundamento dessa relação, assim como, na dimensão da crença (religiosa ou não) e aspectos políticos-ideológicos.

As políticas de vacinação lidam com desafios quais o horizonte pode direcionar com o foco apenas em estratégias de adesão, arriscando vivenciar, em novas emergências sanitárias e/ou necessidade de imunização, cenários de hesitação; ou investir em políticas de aceitação, com fortalecimento da confiança vacinal e, consequentemente, adesão e ampliação da cobertura.

Nesse debate, a categoria acesso precisa ser compreendida para além da dimensão conveniência nos modelos explicativos sobre a hesitação vacinal. Aparentemente, nesse estudo, as barreiras de acesso ao imunobiológico e a hesitação vacinal, para pessoas com o esquema incompleto, se agenciam, o que impossibilita investigá-las separadamente.
